# Water Environmental Capacity Calculated Based on Point and Non-Point Source Pollution Emission Intensity under Water Quality Assurance Rates in a Tidal River Network Area

**DOI:** 10.3390/ijerph16030428

**Published:** 2019-02-01

**Authors:** Lina Chen, Longxi Han, Junyi Tan, Mengtian Zhou, Mingyuan Sun, Yi Zhang, Bo Chen, Chenfang Wang, Zixin Liu, Yubo Fan

**Affiliations:** 1 Key Laboratory of Integrated Regulation and Resource Development on Shallow Lake of Ministry of Education, College of Environment, Hohai University, Nanjing 210098, China; chenlina2001@163.com (L.C.); zy890625@126.com (Y.Z.); grchb@163.com (B.C.); 18852001187@163.com (C.W.); 2 Jiangsu Provincial Academy of Environmental Sciences, Nanjing 210036, China; 3 College of Environment, Hohai University, Nanjing 210098, China; 4 Jiangsu Engineering Consulting Center, Nanjing 210000, China; tanjunyi91@163.com; 5 Academy of Environmental Planning and Design, Nanjing University, Nanjing 210093, China; 18251820679@163.com; 6 Jiangsu Forestry Bureau, Nanjing 210093, China; hhiikkoopp@163.com; 7 College of Science, Hohai University, Nanjing 210098, China; liuzixin888888@icloud.com (Z.L.); f15957199782@163.com (Y.F.)

**Keywords:** tidal river network area, control section, water quality assurance rate, uncertainty, point and non-point source, water environmental capacity

## Abstract

A mathematical model for simulating hydrodynamics and pollutants migration in a tidal river network was constructed, which takes the temporal and spatial distribution of rainfall runoff and non-point pollutants into consideration. Under the design hydrologic conditions of a typical hydrological year, the daily concentration change process for the control section is obtained. Aiming at the uncertainty of hydrology and water quality parameters such as flow direction, flow rate and concentration change in tidal river network area, a statistical analysis method is used to obtain the maximum allowable concentration of pollutants in the control section under the condition of the water quality standard assurance rate of. Then, a formula for calculating the pollutions emission intensity of point and non-point sources is derived. The method was applied to the pollution source control in a typical region like Taihu in China.

## 1. Introduction

The water environmental capacity (WEC) refers to the maximum amount of pollutants that a water body can accommodate under the premise of meeting the water quality standard corresponding to the water environment function. It is also termed the pollution receiving capacity in the literature. There are numerous studies on environmental capacity and pollution capacity, but most of them have focused on a specific river rather than a river network, especially in the tidal river network area [1], yet, the hydrological situation in the tidal river network is very complicated [2], characterized with unsteady flow directions and a large temporal variability of flow rates. Furthermore, even during periods with relatively stable pollution load, the water quality of tidal river networks shows high temporal variability due to variability in hydrodynamic conditions [3,4,5]. Therefore, it is very difficult to determine the environmental capacity of various types of pollutants in a tidal river network area. Several studies have been done regarding this issue.

Considering the reciprocating flow in the tidal river network, based on calculation of the time frequency and discharge for reciprocating flow in one river, the environmental capacities for different flow directions are calculated separately. Finally, the capacity of this tidal river was calculated by the weighted coefficient method, taking the frequency of the occurrence time of reciprocating flow into consideration [6,7]. Although this method considers the difference of the environmental capacity corresponding to the two different flow directions, the weighted average cannot consider the probability of the water quality standard, so for pollutants emission control, the water quality may be in compliance of the water standard at high frequency for a specific river or section, which deviates from the most basic requirements for environmental capacity for water quality stability (a corresponding guaranteed rate). Based on the unsteady model of plain river networks, the water environmental capacity of the river network in the Lucheng District of Wenzhou was estimated by a trial and error method [8], which fully considered the constraint of degree of compliance with the water standards, but it needs repeated trial calculations. This means the calculation efficiency is relatively low and with subjective arbitrariness. Sometimes, the water environmental capacity was calculated annually by month units [9], but it is not conducive to the total pollutant discharge control. Some researchers [10,11] adopted a linear programming method, taking the maximum discharge of pollutants into the river as the objective function, to calculate the environmental capacity by establishing the response relationship between the pollutant discharge of the sewage outlet and the concentration of the control section. However, there are many constraints in a complex river network, and the optimization results may not be representative of the actual situation. Some researchers [12] also used the blind number theory to calculate various possible values of water environmental capacity and its credibility. As a result, these methods are rarely widely accepted due to their shortcomings. Studies on the uncertainty analysis of the water environmental capacity in tidal river networks are relatively deficient.

Therefore, taking a tidal river network area in the Taihu Lake Basin as an example, a method based on mathematical statistics analysis for calculating water environmental capacity in tidal river network areas is proposed. Water quality of the control section and the daily hydrodynamic process was set as the constraints. Time series of the annual variation for the contribution value of present pollutant load were calculated for a given hydrological condition. Finally, we obtained the time series matrix reflecting the mathematical response relationship between the pollutant concentration of the control section and the pollutant load of each pollutant source.

## 2. Methods

### 2.1. Response Relationship of Contaminant Concentration to Influencing Factors in the Control Section

The tidal river network, the flow direction and flow of the water flow may change with the regional hydrological situation. For a specific study period, the time-dependent variation of pollutant concentration in the control section is a linear superposition of concentration contribution time change processes, including the pollutant flux at the inflow boundary, the discharge of point source pollutants, the non-point source pollution load in the river network convergence area and pollutants released from sediments.

When calculating the environmental capacity, a typical and representative hydrological condition of water quality safety should be determined firstly, namely the design hydrological conditions. With the water flow condition as a known parameter, the time-varying process of the control section pollutant concentration can be expressed as a mathematical function:

(1) $$C=F(C_{B},W_{P},W_{N},W_{S})$$

where *C* is the pollutant concentration in the control section, $C_{B}$ is the pollutant concentration of all inflow boundary sections in the controlled area, $W_{P},W_{N},W_{S}$ is the corresponding pollutant release intensity for the point source, non-point source and sediment of the control area, respectively.

### 2.2. Response of Various Pollution Sources to Control Section Pollutant Concentration

#### 2.2.1. Plain River Network Water Flow Model

The river network consists of several single rivers, and the basic equations describing the one-dimensional unsteady flow of open rivers are the one-dimensional Saint-Venant equations:

(2) $$\frac{\partial \;Q}{\partial \;x}+B_{W}\frac{\partial \;Z}{\partial \;t}=q$$

(3) $$\frac{\partial \;Q}{\partial \;t}+2u\frac{\partial \;Q}{\partial \;x}+gA\frac{\partial \;Z}{\partial \;x}-u^{2}\frac{\partial \;A}{\partial \;x}+g\frac{n^{2}\left|u\right|Q}{R^{4/3}}=0$$

where *t* is the time coordinate; x is the space coordinate; *Q* is the flow, m^3^/s; *Z* is the water level, m; *u* is the average flow velocity of the section, m/s; *n* is the roughness; *A* is the cross-sectional area of the flow, m^2^; *B* is the width of the main section, m, *B_W_* is the width of the water surface (including the main width *B* and the additional width that only regulates the storage), m; *R* is the hydraulic radius, m.

The three-stage joint solution method is used to solve the hydrodynamic process of the plain river network. Firstly, each single river is divided into several calculated sections, and the finite difference operation is performed on the Saint-Venant equations on the calculated section to obtain a single river equation—that is, differential equations with independent water level and flow rate as independent variables; Then, according to the connection conditions of the nodes and the boundary conditions, the closed water level equations of each node are formed, and the water level of each node is obtained by solving the equations. Then the water level of each node is returned to the single river equation, and the water level and flow rate of each section of each river are finally obtained.

#### 2.2.2. Water Quality Model of River Network

The governing equation describing the movement and concentration variation of pollutants in a single channel of a river network is a one-dimensional convection-diffusion equation with a source item, the form is as follows:

(4) $$\frac{\partial (AC)}{\partial t}+\frac{\partial (AUC)}{\partial x}=\frac{\partial }{\partial x}(AE_{x}\frac{\partial c}{\partial x})-KAC+S$$

where, *C* is the average cross-sectional concentration of the pollutant, mg/L; *u* is the average flow velocity of the section, m/s; *A* is the area of the section, m^2^; $E_{x}$ is the longitudinal dispersion coefficient, *S* is the pollutant discharge per unit time and unit river length, kg/(s·m); *K* is the pollutant degradation coefficient; *x* is the space coordinate, and *t* is the time coordinate. Using the three-stage joint solution method, the time variation process of pollutant concentration in all nodes and sections of the river network can be obtained.

#### 2.2.3. Time-Varying Pollution Factor Concentration

Using the unsteady water quality mathematical model of river network, the time-varying sequence of pollutant concentration in the control section of the inflow boundary section is reflected in the control section under the designed hydrological conditions (usually typical dry years) can be calculated, and expressed as a column vector:

(5) $$\left\{C_{B}\right\}={\left(c_{B}(1),c_{B}(2),\cdots c_{B}(i),\cdots c_{B}(N)\right)}^{T},i=1,2,\cdots N$$

where *T* represents a vector transposition, *N* is the length of time series, and for a typical hydrological year it can be 365 days, $C_{B}\left(i\right)$ is the concentration response value of the pollutant flow in the inflow boundary of the *i*-th period of the control section, in mg/L. Among them, the concentration of influent pollutants is determined according to the requirements of the water quality management objectives of the inflow boundary of the control area.

Similarly, time-varying sequence of pollutant concentration response in the control section of the river surface source pollution load into the river, expressed as a column vector:

(6) $$\left\{C_{N}\right\}={\left(c_{N}(1),c_{N}(2),\cdots c_{N}(i),\cdots c_{N}(N)\right)}^{T},i=1,2,\cdots N$$

The time-varying sequence of pollutant discharge process in sediments responds to pollutant concentration of the control section, is expressed as another column vector:

(7) $$\left\{C_{S}\right\}={\left(c_{S}(1),c_{S}(2),\cdots c_{S}(i),\cdots c_{S}(N)\right)}^{T},i=1,2,\cdots N$$

Point source pollutants are the main control objects for river network pollution due to the uncertainty of the expected set points and corresponding emissions of the discharge ports. In the current situation, there are more outlets in the control area, generally referring to the current situation of the sewage outlets, calculating time-varying sequence of current sewage outlet and the emissions of current pollution sources (can be assumed to be *Q*) responds to pollutant concentration of the control section, expressed as a column vector:

(8) $$\left\{C_{P}\right\}={\left(c_{P}(1),c_{P}(2),\cdots c_{P}(i),\cdots c_{P}(N)\right)}^{T},i=1,2,\cdots N$$

Since the migration process of pollutants in surface water can be described by the diffusion convection diffusion equation, it corresponds to a first-order dynamic system. Therefore, the principle of linear superposition is satisfied. According to this, the time variation process of the control section pollutant concentration can be expressed by a vector as:

(9) $$\left\{C_{C}\right\}=\left\{C_{B}\right\}+\left\{C_{N}\right\}+\left\{C_{S}\right\}+\left\{C_{P}\right\}$$

### 2.3. Correlation between Control Section Pollutant Concentration and Point Source Pollution Load

From Equation (9), the time-varying process of pollutant concentration in the control section subject to the conditions of each pollution factor under design hydrological conditions (generally using typical hydrological year) can be calculated separately. At present, the environmental capacity is mainly for various types of pollutants discharged from point sources. Therefore, $\left\{C_{B}\right\},\;\left\{C_{N}\right\}$, $\left\{C_{S}\right\}$ in Equation (9) are known column vectors for given hydrological design conditions. Assuming that the point source pollutant emission is *Q* and the point source pollutant discharge subject to the guarantee rate *P* is *W*. Then the control section pollutant concentration can be expressed as the concentration response relationship with each pollution factor:

(10) $$\left\{C_{C,W}\right\}=\left\{C_{B}\right\}+\left\{C_{N}\right\}+\left\{C_{S}\right\}+\frac{W}{Q}\left\{C_{P}\right\}$$

### 2.4. Calculation of Environmental Capacity

Obviously, for a typical design hydrological year, $\left\{C_{C,W}\right\}$ is a time varying sequence of the control section concentration with a duration of *N* = 365 days. It can be regarded as a statistical sample composed of random event values with a capacity of 365. According to the non-deterministic frequency statistical analysis, the concentration of the pollutant corresponding to the guarantee rate P exists objectively in the above statistical sample, and can be assumed to be $\left\{C_{C,W,P}\right\}$. Obviously, in the case of designing pollution factors such as hydrological conditions and watershed sources as a given background value, $C_{C,W,P}=\;\;f\left(W\right)$. Therefore, the solution of the environmental capacity W can be summarized as solving the point source pollutant emission so that the control section pollutant concentration corresponding to the guarantee rate *P* satisfies the water quality target *C_S_*, which can be expressed as the mathematical optimization problem:

(11) $$\left\{ \begin{array}{l} \mathrm{Objective}\;\mathrm{function}:C_{C,W,P}(W)=C_{S}\\ \mathrm{Restrictions}:\left\{C_{C,W}\right\}=\left\{C_{B}\right\}+\left\{C_{N}\right\}+\left\{C_{S}\right\}+\frac{W}{Q}\left\{C_{P}\right\} \end{array}\right.$$

To solve this optimization problem the curve fitting method had been applied. The curve interpolation of the relationship between pollutant concentration and source strength for the control section subject to the design guarantee rate can be used to get the water environmental capacity *W*.

## 3. Application Case and Results

### 3.1. Overview of the Study Area

**Figure 1:**
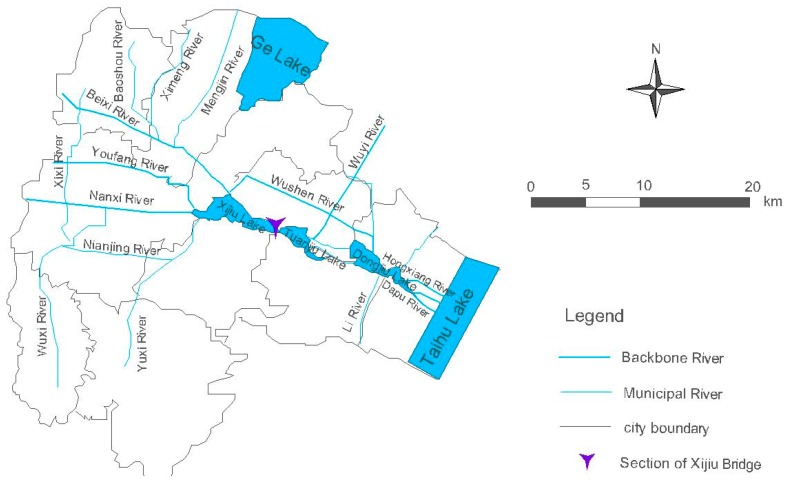
Water System of Study Area.

The study area is located in the Xijiu Lake water system of Yixing City in Jiangsu Province. It is a typical tidal river network belonging to the Nanxi water system of the Taihu Lake Basin. The catchment area includes many rivers such as Nanxi River, Beixi River and Youfang River. The upstream flow through Nanxi River, Beixi River, Youfang River, etc. flow into Xijiu Lake firstly. Finally, the water flows into Taihu Lake after going across the Dongjiu Lake. The section of Xijiu Bridge is the representative section of the water system quality assessment of the water system, and the water quality target is Class III of surface water according to water quality standard in China (GB3838-2002, China). The water system of this study area is shown in Figure 1.

### 3.2. Establishment of the River Network Hydrodynamic Model

#### 3.2.1. Boundary Conditions

According to the long-range annual rainfall data of the basin and the frequency analysis results, the year 2000 was selected as a typical drought year (*p* = 90%) [13,14]. In this case, 13 flow boundaries and four water level boundaries were selected as the boundary conditions for the hydrodynamic boundaries. For the rainfall runoff process the corresponding relationship between the land unit and the inflow section is identified according to the spatial position relationship, and then the runoff of each unit is calculated according to the runoff coefficient. Finally, the temporal process of the land runoff into the river is determined according to the time allocation ratio. We realize the time and space coupling for land runoff and river network confluence.

#### 3.2.2. Parameterization and Validation of Water Model Verification

**Figure 2:**
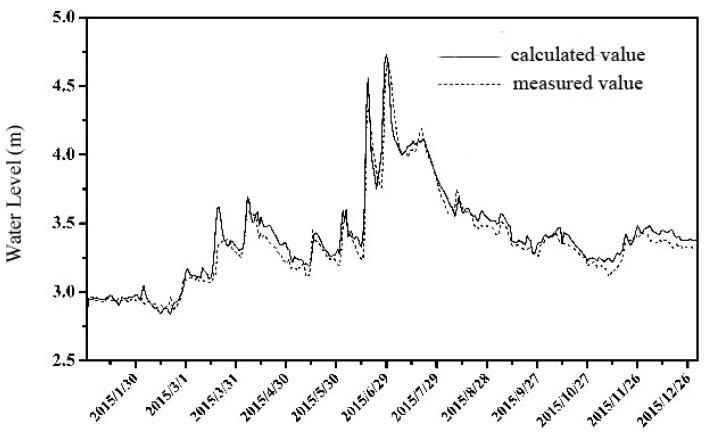
The Verification results of Water Level.

The river roughness is determined by referring to the historical research results for this area. The daily water level of the Xijiu Yixing (West) Station is used for model validation. The comparison between the calculated water level and the measured value is shown in Figure 2. It shows that the calculated water level is in good agreement with the measured value with an average error of 0.03 m.

### 3.3. Establishment of River Network Water Quality Model

#### 3.3.1. Boundary Conditions

According to the target of water quality corresponding to the water function of the adjacent upstream water body at the inflow boundary, the concentration time variation process of the inflow section was given. Here, the second type boundary condition was adopted for the outflow boundary.

#### 3.3.2. Point and Non-Point Source Generalization

There are ten point source sewage outlets in this study area. We set the corresponding relationship between the outlet locations and the related river. Then we identify the corresponding relationship between the land pollution generation and inflow reach [15,16] and the experimental experiential value was studied according to the process of the surface rainfall source concentration of the time-range stream. After determining the time allocation process of land-based sewage into the river [17], finally, we realize the time and space dynamic coupling for the land non-point pollution load and river network pollutant transport.

#### 3.3.3. Parameters and Model Validation

**Figure 3:**
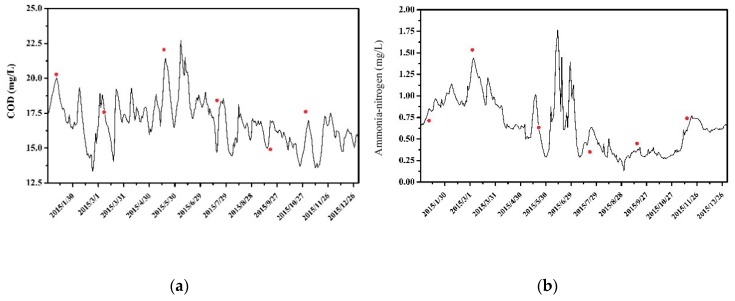
The water quality verification results for (**a**) COD; and (**b**) ammonia nitrogen.

Water quality parameters such as pollutant degradation coefficient and pollutant dispersion coefficient are determined according to the relevant research results in the region. The water quality monitoring data of the Xijiu Bridge section were used for model validation. The comparison between the calculated and measured values of water quality is shown in Figure 3. It shows that the calculated water quality of the Xijiu Bridge section is in good agreement with the measured values. The average relative error for COD is 13.5%, and the relative error for ammonia nitrogen is 16.8%.

#### 3.3.4. Water Environment Capacity and Water Quality Compliance Rate

**Figure 4:**
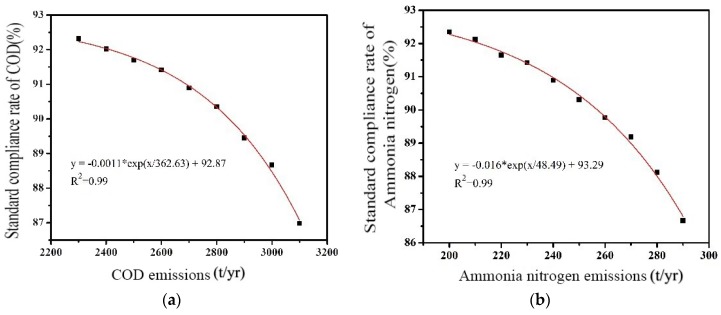
Relationship between the pollutant discharge and guaranteed rate of water quality standard compliance of the control section. (**a**) COD; (**b**) ammonia nitrogen.

Based on the discharge amount of different pollutants, the time guarantee rate of the pollutant concentration for the control section was calculateed by using the curve fitting method. The relationship between the pollutant discharge amount and the control section water quality compliance guarantee rate can be shown as in Figure 4. The environmental capacity of the pollutant subject to any water quality compliance rate can be figured out based on the response. For 90% guarantee rate, water environmental capacity of COD and ammonia nitrogen were determined as 2845.6 t/yr and 257.9 t/yr.

**Figure 5:**
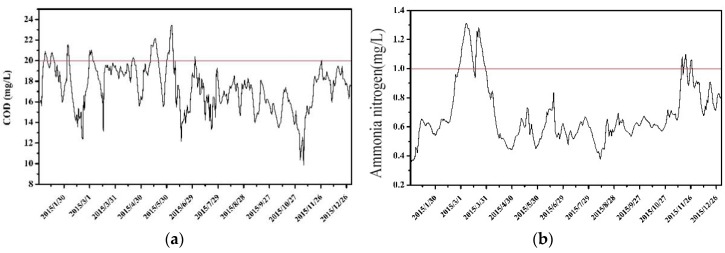
Time series of daily average concentration of pollutants. (the horizontal lines represent the required water quality standard for the control section). (**a**) concentration for COD; (**b**) concentration for ammonia nitrogen.

Then, the allowable point pollutant discharge of each sewage outlet can be set according to the calculated COD and ammonia nitrogen capacity, and used to predict the concentration process of each water quality factor in the typical design hydrological year. The water quality compliance time of each factor meets the set guarantee rate requirement, both higher than 90% for COD and ammonia nitrogen. Among them, the daily average concentration of COD and ammonia nitrogen are shown in Figure 5. The results showed that there 329 days and 330 days in a whole year with the concentration of COD and ammonia nitrogen lower than the water quality standard, respectively. The frequency was 90.1% and 90.4%, respectively.

## 4. Conclusions

As an important technical parameter for regional pollution control, water environmental capacity can be calculated by the formulas for both point sources and non-point sources according to the water quality standard compliance rate, which can overcome the shortcomings of the existing simplified algorithms used for river network environmental capacity calculations. First, the relationship between the pollutant concentration and frequency of the control section should be done by anlyzing the time series of pollutant concentrations. The target pollutant concentration is figured out according to the temporal guarantee rate of the water quality standard compliance of the control section. Then, based on the standard value of water quality and the pollutant concentration value of sewage discharge, the the maximum allowable discharge of various pollutants in the control area can be calculated based on the mathematical model of water flow and quality for river network. The environmental capacity calculation method presented in current research can be extended to non-point source pollution. This method also improves the reliability of calculation and prediction due to associated uncertainties in the related to water quality compliance.

In this study, the convection diffusion equation was used to describe the migration process of pollutants in surface water so that the principle of linear superposition would be suitable for calculating the water environment capacity. Based on this assumption, this method can not only be used for one dimension, like we did in the case, but is also available for two dimension and three dimension modeling, which just need to introduce the corresponding convection diffusion equations for the related dimension direction at the same time.
